# Association of air pollution and weather conditions during infection course with COVID-19 case fatality rate in the United Kingdom

**DOI:** 10.1038/s41598-023-50474-w

**Published:** 2024-01-06

**Authors:** M. Pear Hossain, Wen Zhou, Marco Y. T. Leung, Hsiang-Yu Yuan

**Affiliations:** 1 Department of Biomedical Sciences, Jockey Club College of Veterinary Medicine and Life Sciences, City University of Hong Kong, Kowloon, Hong Kong Special Administrative Region China; 2 WHO Collaborating Centre for Infectious Disease Epidemiology and Control, School of Public Health, Li Ka Shing Faculty of Medicine, The University of Hong Kong, Kowloon, Hong Kong Special Administrative Region China; 3 Laboratory of Data Discovery for Health Limited, Hong Kong Science Park, New Territories, Hong Kong Special Administrative Region China; 4 Department of Atmospheric and Oceanic Sciences and Institute of Atmospheric Sciences, Fudan University, Shanghai, China; 5 School of Marine Sciences, Sun Yat-Sen University, Guangzhou, China; 6 Centre for Applied One Health Research and Policy Advice, Jockey Club College of Veterinary Medicine and Life Sciences, City University of Hong Kong, Kowloon, Hong Kong Special Administrative Regions China

**Keywords:** Infectious diseases, Ecology, Ecology, Environmental sciences, Diseases

## Abstract

Although the relationship between the environmental factors, such as weather conditions and air pollution, and COVID-19 case fatality rate (CFR) has been found, the impacts of these factors to which infected cases are exposed at different infectious stages (e.g., virus exposure time, incubation period, and at or after symptom onset) are still unknown. Understanding this link can help reduce mortality rates. During the first wave of COVID-19 in the United Kingdom (UK), the CFR varied widely between and among the four countries of the UK, allowing such differential impacts to be assessed. We developed a generalized linear mixed-effect model combined with distributed lag nonlinear models to estimate the odds ratio of the weather factors (i.e., temperature, sunlight, relative humidity, and rainfall) and air pollution (i.e., ozone, $N{O}_{2}$, $S{O}_{2}$, $CO$, $P{M}_{10}$ and $P{M}_{2.5}$) using data between March 26, 2020 and September 15, 2020 in the UK. After retrospectively time adjusted CFR was estimated using back-projection technique, the stepwise model selection method was used to choose the best model based on Akaike information criteria and the closeness between the predicted and observed values of CFR. The risk of death reached its maximum level when the low temperature (6 °C) occurred 1 day before (OR 1.59; 95% CI 1.52–1.63), prolonged sunlight duration (11–14 h) 3 days after (OR 1.24; 95% CI 1.18–1.30) and increased $P{M}_{2.5}$ (19 μg/m^3^) 1 day after the onset of symptom (OR 1.12; 95% CI 1.09–1.16). After reopening, many COVID-19 cases will be identified after their symptoms appear. The findings highlight the importance of designing different preventive measures against severe illness or death considering the time before and after symptom onset.

## Introduction

The emergence of COVID-19 has led to an unprecedented number of infections and deaths worldwide. Certain environmental factors, such as weather conditions and air pollution, have been shown to influence disease severity. Knowing the consequence of these factors to which infected individuals are exposed at different infectious stages (e.g., virus exposure time, incubation period, and at or after symptom onset) can potentially help to form guidance on reducing the number of COVID-19 deaths. Unfortunately, the evidence of such differential effects on case fatality rate (i.e., the probability of death after infection) remains largely unknown.

Recent population studies have reported the association between COVID-19 deaths and weather conditions, such as temperature and humidity^1–5^. A colder condition can increase the viability and survival of viruses during disease transmission (for review, see^6,7^), leading to a higher viral load. The viral load has been demonstrated to be associated with disease severity^8–10^. Another possible route to affect disease mortality by temperature and humidity is through modulating immune responses. Studies have found that overreaction of immune responses, such as cytokine storm, triggered by innate immunity, can lead to severe consequences after the infection. Furthermore, the activity of macrophages, which drives innate immunity, has been shown to be associated with temperature^11,12^. This innate defense mechanism generally began after the incubation period^13^. Hence, exposure to environmental factors at or after symptom onset might contribute to such dysregulated innate immunity.

Sunlight duration is a potential environmental risk factor for COVID-19 deaths, as lower vitamin D levels are associated with increased infection severity^14,15^. Sunlight exposure helps synthesize vitamin D, which may reduce COVID-19 severity^16–18^. Presumably, the effect of sunlight likely occurs in the early infectious stages to influence immune response, but no studies have determined at which stages sunlight exposure affects COVID-19 mortality. Sunlight consists of various radiation types, including ultraviolet (UV) radiation. Factors like latitude, altitude, and atmospheric conditions influence UV radiation and sunlight exposure^19^. Longer sunlight duration generally correlates with increased UV radiation exposure, which can be harmful in excess but is necessary in moderation for vitamin D production and a healthy immune system^20^.

Exposure to ambient air pollution is also associated with the transmissibility, population susceptibility, and severity of COVID-19^21–23^. The main components of air pollution are gases and particles such as carbon monoxide (CO), nitrogen dioxide ($N{O}_{2}$), sulphur dioxide ($S{O}_{2}$), ozone (${O}_{3}$), and particulate matter of size $\le 10$ μm ($P{M}_{10}$) and $\le 2.5$ μm ($P{M}_{2.5}$), respectively. As a result, air pollution is considered as the transport of viral particles in the air^24,25^ and within the respiratory tract. By worsening chronic respiratory diseases or modulating immune responses, air pollution could increase the severity of COVID-19^26^. Therefore, it is important to understand the impact of air pollution on disease fatality when they are exposed after the incubation period.

As of September 15, 2020, the United Kingdom (UK) had the third highest number of COVID-19 related deaths worldwide, with 58,032 deaths reported^27^. In response to the initial spread of the virus, a strict social distancing policy was implemented across all UK constituent countries (except Northern Ireland, which adopted the policy 2 days later) on March 26, 2020, to reduce transmission. This lockdown was relaxed on May 13, 2020. Despite the similar lockdown measures being implemented, England was the most significantly affected country among the four UK constituent countries by the end of May 2020^28^.

The present study aimed to investigate two key objectives: firstly, to identify potential risk factors within weather conditions and air pollution that contribute to the likelihood of mortality following COVID-19 infection in the UK; and secondly, to determine the specific infectious stages (e.g., virus exposure time, incubation period, and at or after symptom onset) during which these factors were associated with higher disease severity than other time periods. Owing to the complexity of examining the impact of other control measures, our investigation focused exclusively on the association between environmental factors and COVID-19 case fatality rate (CFR). To ensure the accuracy of our findings, we conducted this study during a timeframe when the intensity of public health and social measures remained relatively stable, and prior to the influence of vaccination or new SARS-CoV-2 variants on mortality rates. This period was defined as March 26, 2020, to September 15, 2020, prior to the first alpha variant was detected in the UK^29^. Utilizing a generalized linear mixed-effect model with distributed lag nonlinear models (DLNM), we were able to assess the risk posed by environmental factors at different stages of infection.

The outcomes of our research hold the potential to inform recommendations for preventive measures aimed at mitigating the severity of the disease, ultimately contributing to a more comprehensive understanding of the interplay between environmental factors and COVID-19 outcomes.

## Material and methods

### Epidemiological data

During the study period from March to September 2020, the case definition for COVID-19 in the UK was primarily based on laboratory confirmation through testing. The real-time reverse transcription polymerase chain reaction (RT-PCR) assay was employed to detect the presence of the SARS-CoV-2 virus in respiratory samples^30^. In March, testing was primarily targeted at hospitalized patients with severe respiratory infections, healthcare workers with relevant symptoms, and other high-risk groups. As testing capacity increased, the criteria for testing expanded to include more individuals with symptoms suggestive of COVID-19.

Deaths due to COVID-19 were defined as deaths in individuals who had a positive test result for the SARS-CoV-2 virus. The UK's Office for National Statistics (ONS) provided data on COVID-19-related deaths, which included deaths where COVID-19 was mentioned on the death certificate as a cause or contributing factor^31^. Data on COVID-19 cases and deaths were obtained from Public Health England and the Office for National Statistics^32^. Laboratory-confirmed cases were identified through RT-PCR testing, as recommended by the World Health Organization^33^.

### Environmental data

Weather data were collected from the European Climate Assessment and Dataset (ECA&D) project^34^. The daily mean temperature was obtained from 120 UK meteorological stations, while mean sunlight duration was available from 24 stations. The temperature data had 1.1% values missing. To address these missing observations, we calculated the average of the temperatures of the previous 7 days to replace the missing values. As relative humidity data were not directly available from ECA&D at the time of data collection, we collected dew point temperatures from the National Oceanic and Atmospheric Administration (NOAA)^35^ to calculate relative humidity following a previous method^36^. Air pollution data, including $CO,$ $N{O}_{2}$, $S{O}_{2}$, ${O}_{3}$, $P{M}_{10}$ and $P{M}_{2.5}$, were obtained from the Air Information Resource provided by the Department for Environment, Food and Rural Affairs, UK^37^. Daily averages of each pollutant were collected for every country during the study period. In cases of missing observations, we employed the same approach as we did for temperature data, whereby the missing value was replaced by the average of the preceding 7 days' readings.

### Back-projection of COVID-19 deaths and estimation of instantaneous CFR

In order to estimate the probability of newly confirmed infected cases who die later due to the infections on a given day, instantaneous case fatality rate (iCFR) was used^38^. One way to calculate iCFR is through a non-parametric back-projection approach to retrospectively adjust the time of death cases^39^. This reduces the possible bias caused by different time points between reporting of cases and deaths when calculating the rate.

**Figure 1:**
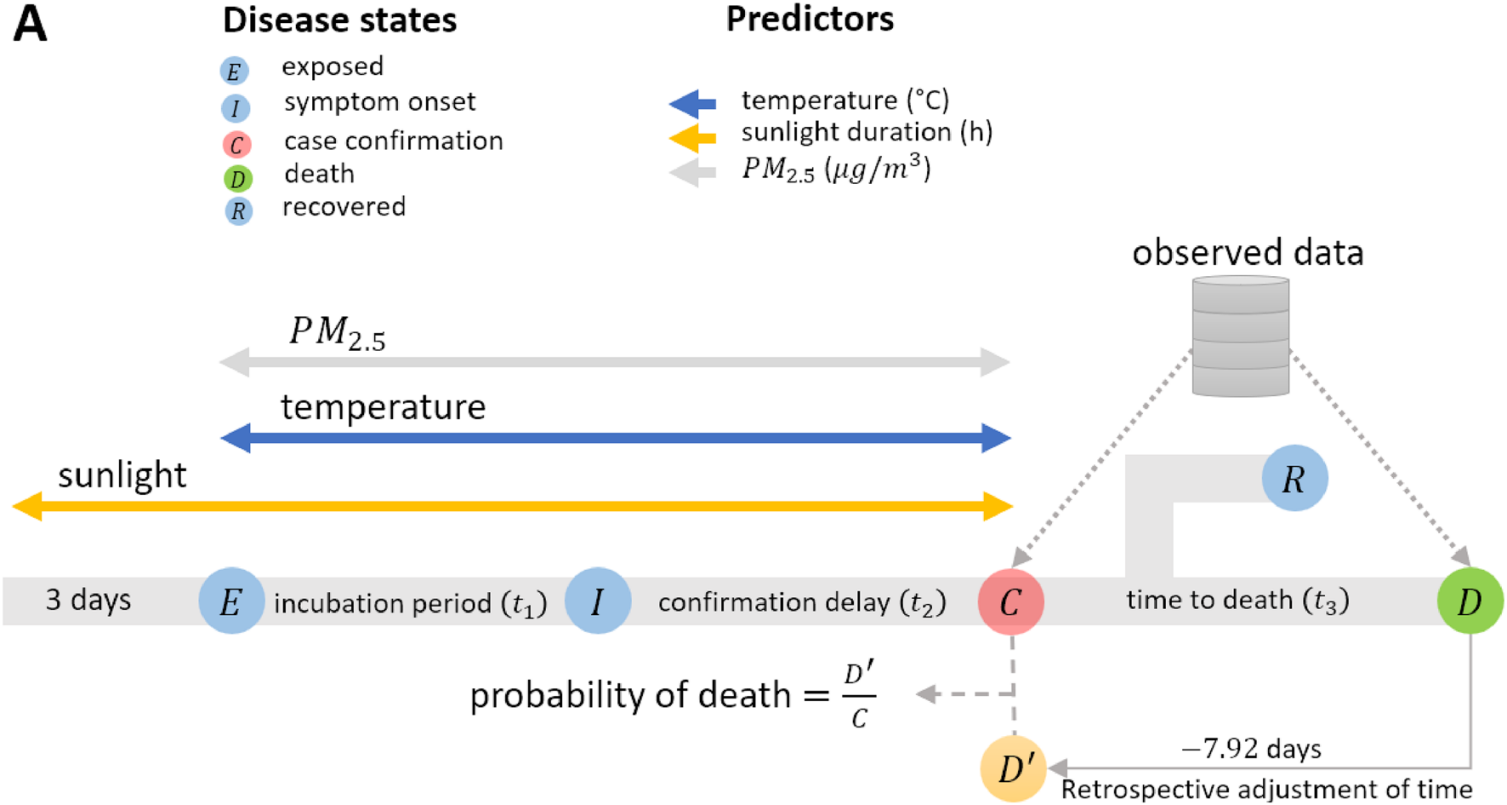
Timeline of disease states and environmental risk factors. $E$,$I$,$C$*,* $R$ and $D$ represent the disease states such as exposed, symptom onset (or the end of incubation period), case confirmation, recovered and reported deaths, respectively. Number of deaths were assumed a subset of infected cases who were reported previous days. Hence, a retrospective adjustment of time was made for estimating deaths who were reported as positive cases at time $t$ using non-parametric back-projection method. These estimated deaths were labeled as $D^{\prime}$, and therefore ${D}^{\prime}\subset C$ for each day. Thus, the iCFR is estimated as the ratio of $D^{\prime}$ and $C$.

We assumed that COVID-19 transmission dynamics appeared in different disease status including as exposed ($E$), symptom onset ($I$), cases confirmation ($C$) and deaths ($D$) (Fig. 1). We assumed the time span between exposure and symptom onset to be ${t}_{1}=5.71$ days (referred as incubation period), and the time between symptom onset and case confirmation to be ${t}_{2}=4.03$ days (referred as confirmation delay). Additionally, the duration between case confirmation and death (time to death) was taken to be ${t}_{3}=7.92$ days. These values were estimated in our recent study^38^. Given the time to death follows a gamma distribution, with a mean of ${t}_{3}=7.92$ days, we retrospectively calculated the actual number of deaths, ($D{\prime}$), which were likely to be members of confirmed cases using an R function backprojNP^40^. Finally, iCFR was calculated as a ratio of $D{\prime}$ and $C$.

### Model formulation

We used a generalized linear mixed-effect model^41^ with DLNM^42^. We adjusted for the effects of relative humidity on the day of exposure to determine whether the iCFR was affected by it on the days when the indexed cases were exposed. As the iCFR represents the probability of death following infection, we modeled the number of deaths out of the total number of cases. A binomial distribution is an appropriate approach for modeling this type of outcome, where the variable of interest is the number of successes (i.e. deaths) out of a fixed number of trials (i.e. cases)^43^. Therefore, we employed a logistic regression model under the Distributed Lag Non-Linear Models (DLNM) framework, which is well-suited for analyzing non-linear relationships between exposure and outcome variables over time.

We assumed the number of deaths, ${D}{\prime},$ follows a binomial distribution with a probability, ${\pi }_{t}^{k}$(the probability of death after infection), among confirmed cases $C$, i.e., ${D{\prime}}_{t}^{k}\sim binomial\left({C}_{t}^{k}, {\pi }_{t}^{k}\right)$, where $k$ indicates a particular location and $t$ represents a day. The model was developed as follows

1 $${\text{log}}\left(\frac{{\pi }_{t}^{k}}{1-{\pi }_{t}^{k} }\right)=\alpha +{\alpha }^{k}+\sum_{i=1}^{3}\sum_{l=0}^{L}{s}_{l}\left({X}_{t,l,i}^{k}; {\beta }_{l,i}\right)+\gamma {H}_{t-\left({t}_{1}+{t}_{2}\right)}^{k}+\delta {W}_{t}+{\varepsilon }_{t}^{k}$$

where ${\pi }_{t}^{k}$ represents the expected iCFR among newly confirmed cases on day $t$ at location $k$ ($k = 1, 2, 3$ or $4$; representing the four countries of the UK), $\alpha$ is the overall intercept of the model and the between-country effects that were considered as the random effects is presented as ${\alpha }^{k}$. ${s}_{l}$ represents a smooth function of the environmental predictor ${X}_{t,l,i}^{k}$ ($i=1, 2, 3$; representing temperature, sunlight duration and $P{M}_{2.5}$) and $l$ represents the lag days from the day of confirmation to the day of exposure. $L$ is the maximum lag, which was defined as the sum of the incubation period and confirmation delay, i.e., $L={t}_{1}+{t}_{2}$ . ${H}_{t-\left({t}_{1}+{t}_{2}\right)}^{k}$ represents the relative humidity on the day of exposure at time $t$ and location $k$. ${W}_{t}$ represents the day of the week on a given day $t$ which allows to adjust for weekly effect of COVID-19 testing whereby more test results are reported on specific days of the week (i.e., first day of the week or weekend). A random error term is represented by ${\varepsilon }_{t}^{k}$. See detailed descriptions in Supplementary Information.

To completely capture the overall impact of weather during the incubation period and confirmation delay, we used a maximum lag of 10 days for temperature and air pollution. For sunlight duration, the time between 3 days before virus exposure and confirmation was considered under the assumption that vitamin D synthesis in individuals can happen before virus exposure and affect the immune response thereafter. The odds ratio of death was calculated using a reference value of each predictor. The linear effect of relative humidity was assessed on the day of exposure because models using distributed lagged effects of relative humidity did not show good fitting results based on Akaike information criterion (AIC).

### Model selection criteria

To identify the best model (best-prediction model) among different combinations of predictors, in a two-stage selection approach, AIC were used in the first stage to choose a set of candidate models using a stepwise selection approach (details in supplementary materials). The models that gave relatively lower AIC were considered the candidate models in the second stage. In the second stage, we compared the model’s output to the observed data. The model that produced the lowest root means square errors (RMSE) was chosen as the best model.

### Ethics approval

The current study did not need any ethical approval since no animal model or organ was not used in this study.

## Results

To estimate the iCFR, we first retrospectively adjusted the daily number of reported deaths to their possible confirmed date and divided this number by the daily number of confirmed cases (Fig. 1). The reported deaths were back-projected to the time of confirmation assuming that infected individuals were died 7.92 days on average after they were confirmed (see Methods).

The iCFR and environmental factors in the UK were analyzed, and we discovered some intriguing trends, as illustrated in Figures S1 and S2. The iCFR was highest across all four countries during the lockdown in the UK, with a gradual decrease over time. Notably, England had the highest iCFR. At the onset of the outbreak, temperature and humidity were low in England and Scotland, but increased later on. Additionally, the concentration of $P{M}_{2.5}$ showed the greatest fluctuation in England and Wales (Figure S2). For more detailed information on these trends and their relationship to CFR, please see the Supplementary Information.

We compared seven models, from a baseline to more complicated models, including different combinations of the weather and air pollution predictors (Table 1). The best model (Model 6), including temperature, sunlight duration and $P{M}_{2.5}$, was selected after showing that the AIC was low and the RMSE of the observed and the estimated values was the lowest than others (Table 2). The model successfully captured the pattern of iCFR in each country (Figure S3).

**Table 1:** Initial model selection.

No	Model	AIC	BIC	Log-likelihood
1	$\alpha +{\alpha }^{k}+\delta {W}_{t}$ Baseline (regional random effect)	2967	2990	-1475
2	$\alpha +{\alpha }^{k}+\delta {W}_{t}+f(S)$ Regional random effect and sunlight duration	650	708	-301
3	$\alpha +{\alpha }^{k}+\delta {W}_{t}+f\left(S\right)+f(T)$ Regional random effect, temperature, and sunlight duration	595	690	-258
4	$\alpha +{\alpha }^{k}+\delta {W}_{t}+f\left(S\right)+f\left(T\right)+f(S{O}_{2})$ Regional random effect, temperature, sunlight duration and sulfur dioxide	581	705	-238
5	$\alpha +{\alpha }^{k}+\delta {W}_{t}+f\left(S\right)+f\left(T\right)+f(P{M}_{10})$ Regional random effect, temperature, sunlight duration and particular matter $(\le 10)$ μm	579	713	-234
6	$\alpha +{\alpha }^{k}+\delta {W}_{t}+f\left(S\right)+f\left(T\right)+f(P{M}_{2.5})$ Regional random effect, temperature, sunlight duration and particular matter $(\le 2.5)$ μm	578	712	-233
7	$\alpha +{\alpha }^{k}+\delta {W}_{t}+f\left(S\right)+f\left(T\right)+f(P{M}_{2.5})+\gamma {H}_{t-\left({t}_{1}+{t}_{2}\right)}^{k}$ Regional random effect, temperature, sunlight duration, particular matter $(\le 2.5)$ μm and humidity	579	715	-232

AIC and BIC represent Akaike information criterion and Bayesian information criteria, respectively. All candidate models were adjusted for the days of the week.

**Table 2:** Model comparison.

Model	Model expression	RMSE
Model 5	$\alpha +{\alpha }^{k}+\delta {W}_{t}+f\left(S\right)+f\left(T\right)+f(P{M}_{10})$ Regional random effect, temperature, sunlight duration and particular matter $(\le 10)$ μm	0.08868
Model 6	$\alpha +{\alpha }^{k}+\delta {W}_{t}+f\left(S\right)+f\left(T\right)+f(P{M}_{2.5})$ Regional random effect, temperature, sunlight duration and particular matter $(\le 2.5)$ μm	0.08555
Model 7	$\alpha +{\alpha }^{k}+\delta {W}_{t}+f\left(S\right)+f\left(T\right)+f(P{M}_{2.5})+\gamma {H}_{t-\left({t}_{1}+{t}_{2}\right)}^{k}$ Regional random effect, , temperature, sunlight duration, particular matter $(\le 2.5)$ μm and humidity	0.08717

The three candidate models (Models 5–7) were compared using predicted results.

*RMSE* root mean squared error.

### Differential risks of environmental factors

**Figure 2:**
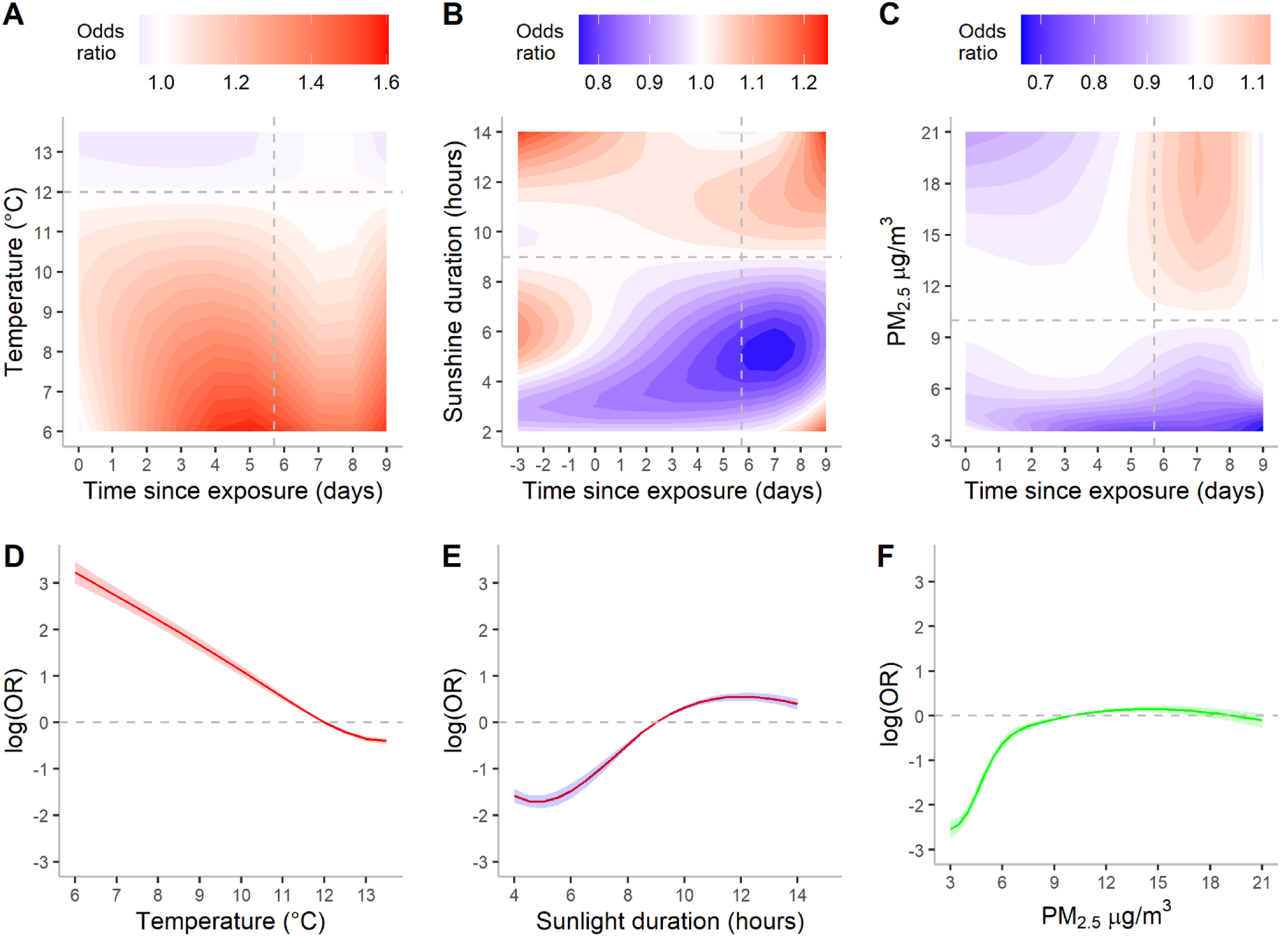
Risk of COVID-19 fatality under different environmental conditions and different time points since virus exposure (**A–C**). (**A**) Temperature, (**B**) sunlight duration and (**C**) particular matter ($P{M}_{2.5}$). The vertical dashed lines in A, B, C represent the date of symptom onset (on day 5.71 since exposure). Therefore, time between 0 and 5.71 days represents the incubation period. Odds ratio was estimated with respect to the reference value (horizontal lines in A, B, C) of each predictor. Reference value for temperature was ${12}^{\circ }C$, sunlight duration 9h and ${PM}_{2.5} \;10 \; \mu g/{m}^{3}$*.* Cumulative effects of environmental factors on the odds ratio of COVID-19 mortality (**D**–**F**). Horizontal dashed lines represent the baseline odds ratio at the reference values of the environmental predictors. The shaded regions represent 95% confidence interval of the log transformed odds ratio. The overall effect of temperature and $P{M}_{2.5}$ was estimated for the duration between virus exposure and the confirmation day, whereas the cumulative effects of sunlight duration was estimated from three days prior to the virus exposure to the case confirmation day.

Using the best model obtained in the preceding section, we assessed the differential effects of temperature, sunlight duration, and $P{M}_{2.5}$ throughout the course of infection. Compared to the reference temperature of ${12}^{\circ }C$, low temperatures between 6 and 11 °C before the incubation period were associated with a higher risk (measured by odds ratio) of death (Fig. 2A). A temperature of 6 °C at 5 days after the exposed to virus gave a maximum OR of 1.59 (95% CI 1.52–1.63). When temperatures were more than 11 °C, the death risk became lower both during and after the incubation period. Whether infected cases changed their behaviors, such as staying indoors more during those very cold days is unknown.

Furthermore, we found that the relationship between sunlight duration and fatality was distinctly different before and after the estimated symptom onset (i.e., during and after the incubation period) (Fig. 2B). The duration of sunlight after the appearance of symptoms appeared to be more harmful. Prolonged duration of sunlight (11–14 h) about 3 days after symptom onset was associated with a greater risk of death (OR 1.24; 95% CI 1.18–1.30). However, the shortened duration of sunlight, in contrast, showed a beneficial effect during the incubation period or earlier.

$P{M}_{2.5}$ showed a significant impact after symptom onset, such that a higher $P{M}_{2.5}$ of 11–21 $\mu g/{m}^{3}$ was associated with a higher OR of death (Fig. 2C). The maximum OR was observed 1 day after the symptom onset with a value of 1.12 (95% CI 1.09–1.16) when $P{M}_{2.5}$ reached 19 $\mu g/{m}^{3}$, compared with the reference $(P{M}_{2.5}=10 \mu g/{m}^{3} ).$ The OR of these factors at specific infectious stages was described in the section *the effects of weather on the iCFR at specific time points* in Supplementary Information (see Figure S4).

### Cumulative and marginal effects of environmental factors

The cumulative effects of temperatures and $P{M}_{2.5}$ were estimated for the duration between virus exposure and 3 days after symptom onset (or incubation period), whereas the cumulative effects of sunlight duration were estimated from 3 days prior to the virus exposure to 3 days after symptom onset (Fig. 2D-F).

Overall, the cumulative effects (measured by log (OR)) of low temperatures (6–11 °C) were higher (Fig. 2D). Sunlight durations of 10–14 h was significantly associated with a higher OR, while lower sunlight durations of < 9 h appear to be protective (Fig. 2E). The cumulative effects of low $P{M}_{2.5} (3-9\; \mu g/{m}^{3} )$ were significantly low and the effects of high $P{M}_{2.5} (11-17\; \mu g/{m}^{3} ).$ (11–17 μg/m^3^) were substantially high (Fig. 2F), suggesting a positive relationship between iCFR and $P{M}_{2.5}$. While comparing the environmental predictors, the cumulative effect of temperatures showed a larger variation than other predictors.

**Figure 3:**
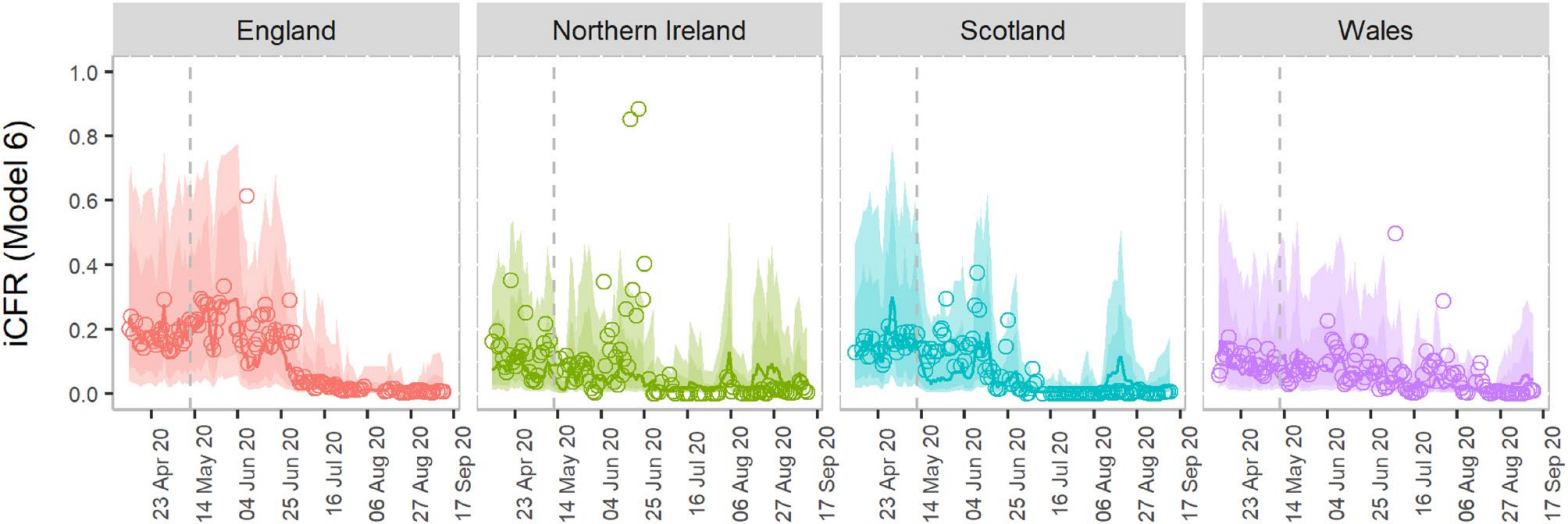
Sensitivity analysis of the best model (Model 6). The panels show the model's prediction results until mid-September, the time when the first alpha variant has been detected in the UK. The points in each subplot represent the instantaneous CFR (iCFR) estimated using the back-projection method for each date. Solid lines represent the CFR estimated using the best-prediction model. The shaded regions indicate pointwise 75% and 95% prediction intervals, respectively. The vertical dashed lines represent the day until which we train the data in the model, whereas on the right side of the line are tested data.

Finally, we validated the best model (Model 6) by predicting future iCFR (see Methods). The model was able to capture the trend among all countries. 81% of observed data were successfully predicted within 95% confidence interval (Fig. 3).

## Discussion

Studies have revealed that the mortality rate or CFR of COVID-19 is influenced not only by the virulence of the SARS-CoV-2 virus but also by environmental factors like weather and air pollution^1–5,21,44–48^. However, the extent to which these factors affect different stages of infection remains unclear. To address this gap, we employed a distributed lag nonlinear model in this study, which allowed us to investigate the lag effects of environmental variables on individual infectious statuses of infected cases, including the exposure period, incubation period, and symptomatic period. Our findings indicate that adopting different precautionary measures before and after symptom onset is crucial. Specifically, our results suggest that exposure to a particular temperature range, such as 6–11 °C, may increase the risk of COVID-19 mortality during or after the incubation period. This could be due to the potential impact of cold temperatures on the immune responses of COVID-19 patients^13,38^.

Sunlight appeared to have an important role in mortality. It affects the production of vitamin D^49^. Vitamin D deficiency results in impaired immune function, which can increase the risk of infectious diseases, such as those caused by respiratory viruses^50^. Recent studies showed that there are no significant differences in hospital mortality between the vitamin D3 group and the placebo group^51^. A systemic review, however, investigated seven out of nine studies indicated that the lack of vitamin D greatly impacts the severity and death of COVID-19^52^. Prolonged exposure to sunlight has been found to inactivate SARS-CoV-2^53,54^, resulting in a reduced risk of infection or disease severity. However, whether prolonged exposure to sunlight may also suppress the proper functioning of the immune system is unknown, especially after the incubation period^55^. Our findings suggest the possible preventive effects of sunlight duration on the disease severity of COVID-19 during and after the incubation period.

A mixed conclusion has been produced in recent studies regarding the connection between PM_2.5_ air pollution and COVID-19 infection and mortality rates. Various investigations have examined the relationship between air pollution and the severity and mortality of COVID-19 at both ecological and individual levels^21,48,56^. Some studies have found that the incidence and fatality of the disease increase significantly with higher concentrations of PM_2.5_^57,58^. For instance, research conducted in the United States has linked long-term exposure to air pollution with COVID-19 mortality^44,45,48^. Similarly, a study in England using high-resolution geographical data also found that long-term exposure to NO_2_ and PM_2.5_ increased the risk of COVID-19 mortality^47^. However, a study conducted in London, United Kingdom, did not find the association. While an initial analysis indicated that long-term exposure to air pollutants was associated with a higher risk of COVID-19 death among individuals who tested positive for the virus, no association was found when more confounding factors were considered^59^. Another study conducted in the UK did not find the association between air pollution and COVID-19 hospitalizations or deaths after adjusting for relevant variables^60^. We found that $P{M}_{2.5}$ exposed after symptom onset might have a significant negative impact on CFR. 

Recently, many studies have been conducted on the association between ambient temperatures and deaths where the inverse relationship was justified^3,38,61,62^. Our study is also consistent with their findings that lower temperatures were associated with a higher risk of mortality. However, we were not able to exclude the possible influences of behavioral changes in the population during a very cold time. In cold temperatures, people usually stay more indoors and may be affected by the room temperatures rather than ambient temperatures. Although our study did not incorporate indoor temperature, a previous study suggested that indoor temperature was strongly correlated with outdoor temperature^63^. Therefore, the overall pattern of the risk of temperature might likely be similar even when indoor temperature is used.

Furthermore, the potential for exposure misclassification may arise from utilizing average air pollution data for each country. This approach may not accurately capture variations in exposure levels between urban and rural areas or among different regions within each country. For example, exposure to pollutants in cities is likely to be higher compared to rural areas, and exposure to specific air pollutants in Scotland generally exhibits lower pollution levels than in England.

The study's data collection was limited to the initial lockdown period in the UK (March to September 2020) with consistent interventions, during the winter season characterized by colder temperatures and reduced sunlight. However, the study did not account for potential changes in population behavior and indoor environments, such as the use of heaters, which could affect environmental factors. Moreover, the non-parametric back-projection approach used to estimate the time of deaths assumed a constant distribution of time from confirmation to death across all cases, leading to a potential bias in exposure timing in relation to infection and mortality. Although this approach has been widely used in previous studies^39^, further research is necessary to better comprehend the effects of environmental exposures on disease severity.

## Conclusions and future directions

For example, how to maintain proper environmental conditions, such as indoor temperature, sunlight, and air quality, during quarantine or home-isolation periods to reduce the probability of death is largely unknown. After many restrictions were lifted in many countries, people with COVID-19 symptoms are advised to get tested and self-isolate. Understanding the relationship between these environmental factors and iCFR especially after symptoms appear provides important suggestions for reducing the number of severe cases.

However, it is also important to acknowledge that England experienced atypical anti-cyclonic conditions during the initial phase of the pandemic^64^, characterized by calm winds and sunny days. These weather patterns might have coincided with the onset of the pandemic by chance, and it is necessary to consider this factor as a potential confounding variable. Therefore, caution should be exercised in attributing causality to the associations observed specially for the $P{M}_{2.5}$ concentration. To further validate and enhance the robustness of these findings, it is crucial to conduct future studies that encompass multiple waves of COVID-19 and explore different geographical regions. Such studies would help test the consistency of the observed associations and provide a more comprehensive understanding of the relationship between weather patterns and the progression of the pandemic.

### Supplementary Information

Supplementary Information.

## Data Availability

The data on COVID-19 cases and deaths and air pollution are publicly available in GOV.UK (https://www.gov.uk/coronavirus) and Air Information Resource in the Department for Environment Food and Rural Affairs, UK (https://uk-air.defra.gov.uk/) respectively. The weather data is also available in the public repository European Climate Assessment and Dataset (https://www.ecad.eu/).
